# Costs of multiple sclerosis in Panama from societal, patient perspectives and health-related quality of life

**DOI:** 10.1371/journal.pone.0204681

**Published:** 2018-10-10

**Authors:** Fernando Gracia, Mario Larreategui, Gaudencio Rodríguez, Aaron Benzadón, Michelle Ortiz, Divian Morales, Claudia Domínguez, Rosa Enith Carrillo, Carlos Valderrama, Luís Lizán, Blas Armién

**Affiliations:** 1 Universidad Interamericana de Panamá, Panamá City, Panamá; 2 Hospital Santo Tomás, Panamá City, Panamá; 3 Instituto Conmemorativo Gorgas de Estudios de la Salud, Panamá City, Panamá; 4 Hospital Anita Moreno-Ministerio de Salud, Los Santos, Panamá; 5 Dirección Nacional de Finanzas-Ministerio de Salud, Panamá City, Panamá; 6 Complejo Hospitalario Metropolitano Arnulfo Arias Madrid-Caja de Seguro Social, Panamá City, Panamá; 7 Hospital Regional Rafael Hernández-Caja de Seguro Social, Chiriquí, Panamá; 8 Outcomes’10, Universitat Jaume I, Castellón, Spain; University of South Florida, UNITED STATES

## Abstract

The purpose of this work is to estimate the costs associated with managing patients with MS in Panama and evaluating the impact of the disease on their health-related quality of life (HRQoL). Multicentric observational, retrospective, cross-sectional study. The costs were estimated from societal and patient perspectives and expressed in USD, 2015. The focus of the study is based on prevalence and on a “bottom-up” approach. To estimate the total cost per patient, annual reported use for each resource was multiplied by its unit cost. To evaluate HRQoL, patients completed the EQ-5D-3L questionnaire. 108 patients took part in the study. 82.41% were women with 44.78 (SD: 12.27) years. 61.11% presented mild (EDSS = 0–3.5), 25.93% moderate (EDSS = 3.5–6) and 12.96%, severe disability (EDSS≥6.5). The mean annual cost from the patient’s perspective was estimated at 777.99 USD (SD: 1,741.45) per patient. The mean cost from a societal perspective was estimated at 23,803.21 USD (SD: 13,331.83) per patient. Disease-modifying therapies (DMT) accounted for the main component of the cost. A deterioration in HRQoL was observed as the disease advances and as disability increases, with mobility and usual activities being the areas most affected by its progression. From both perspective, the cost per MS patient in Panama is high. In addition to the high economic impact, MS also exerts a negative impact on patient HRQoL, which increases as the disease advances.

## Introduction

Multiple sclerosis (MS) is a multifocal demyelinating disease that follows a chronic and disabling course and results in a progressive neurodegeneration caused by an autoimmune response in genetically predisposed subjects [1]. Although MS can be detected at any age, it is usually diagnosed during early adulthood. The average age of MS onset is 30 [2].

MS is one of the most frequent neurological disorders worldwide and in many countries represents the primary cause of disability in young adults [3]. Data on prevalence in Latin America are scarce [4] and it is estimated to vary between ≥ 5 to 25 cases per 100,000 inhabitants [5]. In recent information, the estimated prevalence of MS in Central America varied between 0.9–8.9 and in the Spanish Caribbean Region oscillated between 1.6–77.7 cases per 100,000 inhabitants [6]. In Panama, the crude estimate prevalence in 2016 is 8.9 cases per 100,000 inhabitants, being 74.9% female [6].

Given the young age at which the disease appears and its degenerative nature, which entails progressive disability, the economic burden associated with MS is considerable. Likewise, bearing in mind that MS affects young adults in the most productive stages of life, the impact of the disease on the health-related quality of life (HRQoL) and the work productivity of patients and their caregivers is considerable, which in turn imposes a significant social burden.

Studies performed in Europe show that the economic impact of MS increases as the disability associated with the disease advances and that the distribution of costs also changes [7–10]. Hence, while for patients with mild disability the cost of drugs is the main component of the patient’s total outlay, as disability increases, the non-medical indirect and direct costs become more significant [7–10]. Recently, a cross-sectional study conducted in 16 European countries [11–26] confirmed the results obtained in previous studies. From societal perspective mean costs of mild disease in European countries was 22,800€ PPP (€,2015 adjusted for purchasing power parity), increasing to 37,100€ PPP in moderate and to 57,500€ PPP in severe MS. Proportion of total cost related to healthcare decrease with disease severity: from 68% in mild MS to 26% in severe MS [11–26].

Information on the cost of the disease in Latin America is very limited, most studies having been performed in Brazil, Colombia and Argentina [5,27,28]. In Brazil, the annual direct cost was estimated at 19,012.32 USD (SD: 10,465.96), with no significant differences identified according to the patient’s disability [Expanded Disability Status Scale (EDSS) in mild (0–3), moderate (4–6.5) and severe MS (≥7)] [28]. In Colombia, differences were detected according to the patient’s disability, with the total cost, from the perspective of the third-party payers, for a patient EDSS 3–5.5 of 25,713 USD, and for a patient with EDSS 8–9.5 of 10,543 USD [29]. The estimated costs of MS in Argentina from a societal perspective also differed with the severity of the disease. A patient with EDSS ≥ 7 bore the highest cost, which could amount to 50,712 USD per patient. In Latin America, as in Europe, the cost of drugs accounted for the highest proportion of the total cost, mainly among those patients with a lower degree of disability [28,29].

There are currently no data available on the economic burden of MS in Panama. Data from cost-of-illness (COI) studies can be very useful in making any economic assessment aimed at reducing uncertainty and optimising design and decision-making in healthcare policy. The objective of this study is to estimate the costs associated with the management of MS patients in Panama, from both societal and patient perspectives. A secondary objective is to evaluate the impact of the disease on patient HRQoL.

## Materials and methods

### Study design and patient assessment

This is nationwide observational, retrospective, cross-sectional study of Panama.

The costs were estimated from societal and from patient perspectives and the results expressed in United States dollars from 2015 (USD, 2015). The study is prevalence-focused, which means that the moment the disease appeared is not taken into consideration, but rather its prevalence in the study period. To gather the information, a design based on the “bottom-up” costing technique was used whereby information is obtained directly from patients by means of a survey [30].

Patients included in the study were selected at random from the database of the *Registro Panameño de Esclerosis Múltiple*. These were required to comply with the following inclusion criteria: to be at least 18 years old, to have been diagnosed with MS in accordance with the McDonald criteria [31], to have been monitored for the disease for a year and have provided written consent for their participation in the study. During the data collection period (October 2015- July 2016) the selected patients answered an e-questionnaire using a personal digital assistant (PDA). The interviews were conducted in the regions where the neurology services are located, in four public and six private centres of three provinces: Panama, Chiriqui and Herrera this has not change since 2009 [32]

The study protocol was approved by the Bioethics Research Committee of the *Instituto Conmemorativo Gorgas de Estudios de la Salud* (ICGES—Gorgas Commemorative Institute of Health Studies) in Panama (N° 1167/CBI/ICGES/15).

### Data collection

The MS questionnaire was adapted from the questionnaire employed in a dengue cost survey performed in Panamá [33] and Puerto Rico [34]. The questionnaire was revised by clinical and economic national and international expert. Patients were interviewed by a trained health professional (nurse and medical doctor) using a standardized survey instrument. All study participants were interviewed once or twice in person, either at the patient’s house or at the MS clinic. Each interview lasted about 45 minutes.

The questionnaire issued to patients gathered information on 1) the patient’s sociodemographic (age, year of birth, gender, family situation and education and socioeconomic status) and clinical characteristics: first symptom and the year of diagnosis, MS flare-up in the last year, type of EM, and EDSS (mild, moderate and severe) [35]; 2) the use of healthcare resources (public and private sector) and costs borne by the patient and/or reimbursed by their private insurance associated with each item in the management of the disease during the year prior to the interview; 3) productivity losses of patients and their relatives arising from MS during the year prior to the interview; and 4) their HRQoL at the time of the interview.

Information on the use of resources and costs included healthcare and non-healthcare related data only related to MS. Healthcare resources and costs covered consultations, examinations and treatment arising from in-patient stays and outpatient services; immunomodulatory (interferon beta-1a and -1b (n = 78), fingolimod (n = 13), natalizumab (n = 5), teriflunamida (n = 3) and without treatment (n = 9)) and symptomatic drug treatment of MS, as well as the treatment used in relapses; and additional laboratory examinations and tests. Non-healthcare resources included information on transportation, food and accommodation of the patient/family members arising from in-patient stays and outpatient services.

In order to estimate losses of productivity, data on work lost because of the disease were collected both by the patient and by family members. In addition, long-term sick leave and early retirement by EM were also included.

Lastly, to evaluate the HRQoL of the patients at the time of the interview, they completed the EuroQoL 5 Dimensions Questionnaire—EQ-5D-3L. The EQ-5D-3L questionnaire is a generic tool on HRQoL that measures the extent of HRQoL deterioration using 5 dimensions: mobility, self-care, usual activities, pain/discomfort and anxiety/depression. It also includes a visual analogue scale that measures patient-perceived HRQoL (EQ-VAS). There are three levels of response for each dimension (no problems, some problems, extreme problems). In the EQ-VAS patients indicate their overall current status of health on a scale of 0 (worst imaginable health status) to 100 (best imaginable health status) [36].

### Health care and health services use

#### Cost estimation

The direct healthcare cost was deducted from the sum of the costs incurred by consultations, examinations and treatments during hospitalizations and in outpatients’ services. The direct non-healthcare cost was estimated from the sum of the cost incurred by transport, food and accommodation during hospitalizations and in outpatients’ services.

In the patient’s perspective, all the direct healthcare and non-healthcare costs assumed by the patient were included. The estimated reimbursement for health insurance was deducted from the sum of these costs.

To estimate the costs from the perspective of society the costs considered were the direct healthcare and non-healthcare costs, and the indirect costs associated with losses of productivity (short-term losses of productivity of patients and their relatives and long-term sick leave and early retirement).

To estimate the costs of drugs from this perspective, the cost assumed by public centres (Santo Tomas Hospital of the Ministry of Health) or payable by “Caja de Seguro Social” were considered. In order to estimate the indirect costs, lost days of work were multiplied by the cost of one day of work (according to the Panamanian minimum wage of 17.17 USD) [37].

In all the cost estimates, it was assumed that patients who did not reported data in resources or productivity losses, their costs were 0. So, the calculation of the statistics was made for the total of the sample.

### Sample size and statistical analysis

The sample size was determined on the basis of the adult population of Panama in the year 2015 (2,674,223 inhabitants) [38], the prevalence of MS (5.2/100,000 inhabitants) [32], assuming maximum uncertainty, a confidence level of 95% and precision of 6%. Using these data, the minimum necessary sample size was estimated to be 92 patients.

STATA v.14 statistical software was used for statistical analysis [39]. To describe the quantitative variables, centrality and dispersion statistics were used (mean, standard deviation–SD-). Qualitative variables were described using relative and absolute frequencies. The estimated costs were described with centrality and dispersion measurements in each of the perspectives, and in the subgroups according to EDSS classification.

## Results

### Patient characteristics

A total of 110 patients met the study’s inclusion criteria. Of these, 2 were eliminated from the final analysis as there were inconsistencies in the reported costs. The final sample was composed of 108 patients.

82.41% of the patients were women with an average age of 44.78 (SD: 12.27) years. On the basis of the EDSS, 61.11% (66/108) of participants had a mild disability (EDSS = 0–3.5), 25.93% (28/108) a moderate disability (EDSS = 3.5–6) and the remaining 12.96% (14/108) had a severe disability (≥ 6.5). This is proportional to the distribution of patients in the Panamanian MS Registry (n = 351) where at least 70% have an EDSS 0–3.5 at time of diagnosis 2005–2015 [6].

The main sociodemographic and clinical characteristics of study participants are shown in Table 1. Note that none of the patients with severe disability was in active employment.

**Table 1:** Patients’ demographics and clinical characteristics according to disability level.

	EDSS 0–3.5 (n = 66)	EDSS 3.5–6 (n = 28)	EDSS ≥6.5 (n = 14)	TOTAL (n = 108)
**Gender [n (%)]**	** **	** **	** **	** **
Male	6 (9.09)	10 (35.71)	3 (21.43)	19 (17.59)
Female	60 (90.91)	18 (64.29)	11 (78.57)	89 (82.41)
**Age (years) [mean (SD)]**	42.26 (11.67)	48.61 (9.43)	50.00 (14.89)	44.91 (11.98)
** Marital status [n (%)]**	** **	** **	** **	** **
Single	23 (34.85)	3 (10.71)	4 (28.57)	30 (27.78)
Married	38 (57.58)	21 (75.00)	6 (42.86)	65 (60.19)
Divorced	2 (3.03)	3 (10.71)	3 (21.43)	8 (7.41)
Widowed	2 (3.03)	1 (3.57)	1 (7.14)	4 (3.70)
**Working status [n (%)]]**	** **	** **	** **	** **
Government employee	21 (31.82)	6 (21.43)	0 (0.00)	27 (25.00)
Non-government employee	15 (22.73)	6 (21.43)	0 (0.00)	21 (19.44)
Self-employed	5 (7.58)	1 (3.57)	0 (0.00)	6 (5.56)
Boss	0 (0.00)	1 (3.57)	0 (0.00)	1 (0.93)
Retired	1 (1.52)	1 (3.57)	0 (0.00)	2 (1.85)
Student	5 (7.58)	0 (0.00)	1 (7.14)	6 (5.56)
Retired due to MS	9 (13.64)	8 (28.57)	6 (42.86)	23 (21.30)
Long-term sick leave	7 (10.61)	4 (14.29)	7 (50.00)	18 (16.67)
Others	3 (4.55)	1 (3.57)	0 (0.00)	4 (3.70)
**Annual income [n (%)]**				
<10,000 USD	23 (34.85)	6 (21.43)	7 (50.00)	36 (33.33)
10,000–40,000 USD	27 (40.91)	16 (57.14)	2 (14.29)	45 (41.67)
40,000–70,000 USD	10 (15.15)	3 (10.71)	1 (7.14)	14 (12.96)
70,000–100,000 USD	2 (3.03)	1 (3.57)	0 (0.00)	3 (2.78)
>100,000 USD	3 (4.55)	0 (0.00)	1 (7.14)	4 (3.70)
**Private health insurance [n (%)]**	18 (27.27)	8 (28.57)	3 (21.43)	29 (26.85)
**Time since diagnosis (years) [mean (SD)]**	8.62 (4.88)	10.72 (6.80)	11.67 (7.36)	9.59 (5.85)
**MS flare-up in the last year [n (%)]**				
None	48 (72.73)	17 (60.71)	8 (57.14)	73 (67.59)
1 flare-up	11 (16.67)	5 (17.86)	2 (14.29)	18 (16.67)
2 flare-ups	4 (6.06)	2 (7.14)	2 (14.29)	8 (7.41)
3 flare-ups	2 (3.03)	2 (7.14)	0 (0.00)	4 (3.70)
4 flare-ups	1 (1.52)	2 (7.14)	0 (0.00)	3 (2.78)
**HRQoL (EQ-5D-3L) [n (%)]**				
Mobility				
No problems	**43 (65.15)**	5 (17.86)	0 (0.00)	48 (44.44)
Some problems	23 (34.85)	**23 (82.14)**	3 (21.43)	49 (45.37)
Confined to bed	0 (0.00)	0 (0.00)	**9 (64.29)**	9 (8.33)
Self-care				
No problems	**62 (93.94)**	**18 (64.29)**	3 (21.43)	83 (76.85)
Some problems	4 (6.06)	10 (35.71)	5 (35.71)	19 (17.59)
Unable	0 (0.00)	0 (0.01)	**6 (42.86)**	6 (5.56)
Usual activities				
No problems	**46 (69.70)**	8 (28.57)	1 (7.14)	55 (50.93)
Some problems	19 (28.79)	**20 (71.43)**	4 (28.57)	43 (39.81)
Unable	1 (1.52)	0 (0.00)	**8 (57.14)**	9 (8.33)
Pain/discomfort				
No pain or discomfort	25 (37.88)	3 (10.71)	1 (7.14)	29 (26.85)
Moderate pain or discomfort	**37 (56.06)**	**24 (85.71)**	**11 (78.57)**	72 (66.67)
Extreme pain or discomfort	4 (6.06)	1 (3.57)	2 (14.29)	7 (6.48)
Anxiety/depression				
Not anxious or depressed	**31 (46.97)**	**13 (46.43)**	**5 (35.71)**	49 (45.37)
Moderately anxious or depressed	**30 (45.45)**	**12 (42.86)**	4 (28.57)	46 (42.59)
Extremely anxious or depressed	5 (7.58)	3 (10.71)	**5 (35.71)**	13 (12.04)
**EQ-5D-3L VAS [Mean (SD)]**	76.49 (18.40)	66.61 (16.22)	52.64 (21.22)	70.79 (19.87)

Insofar as the HRQoL of participants is concerned, deterioration in the HRQoL was observed as the disease and the disability advanced. The dimensions of HRQoL most affected by the progress of the disability are mobility and usual activities. The dimensions of pain/discomfort and anxiety/depression are affected from the initial stages of the disease (Table 1).

### Cost estimation from the patient’s perspective

The mean annual cost from the patient’s perspective was estimated at 777.99 USD (SD: 1,741.45) per patient. Direct healthcare costs amounted to 715.07 USD (SD: 1,751.39) representing the 92% of total cost, while direct non-healthcare costs amounted to 75.32 USD (SD: 199.71) per patient (Table 2). According to disability level, the cost was higher for patients with moderate disability (973.13 USD/patient), followed by patients with mild disability (799.96 USD/patient) and, lastly, patients with severe disability (284.11 USD/patient).

**Table 2:** Cost estimation from a patient perspective according to disability level.

	EDSS 0–3.5 (n = 66)	EDSS 3.5–6 (n = 28)	EDSS ≥6.5 (n = 14)	TOTAL (n = 108)
	Mean (SD)	Mean (SD)	Mean (SD)	Mean (SD)
***Direct healthcare costs***				
Consultations, examinations and treatments (in hospital)	0.00 (0.00) USD	25.18 (92.75) USD	0.00 (0.00) USD	6.52 (47.89) USD
Consultations, examinations and treatments (in outpatients’ services)	337.26 (1119.33) USD	555.07 (1377.01) USD	91.07 (191.92) USD	361.81 (1224.18) USD
Additional examinations and tests	181.95 (543.38) USD	129.46 (359.63) USD	51.36 (123.34) USD	151.42 (464.61) USD
Drug treatment of flare-ups	25.74 (98.36) USD	52.68 (151.28) USD	0.00 (0.00) USD	29.39 (109.16) USD
Disease-modifying therapies (DMT)	164.41 (742.74) USD	154.43 (466.30) USD	71.43 (267.26) USD	149.77 (632.15) USD
Symptomatic drug treatment	14.64 (48.96) USD	19.25 (54.90) USD	17.14 (39.01) USD	16.16 (49.05) USD
**Total direct healthcare costs**	**724.00 (1927.05) USD**	**936.07 (1724.18) USD**	**231.00 (383.41) USD**	**715.07 (1751.39) USD**
***Non-healthcare direct costs***	** **	** **	** **	** **
Transport, food and accommodation (in hospital)	0.00 (0.00) USD	3.93 (11.96) USD	0.00 (0.00) USD	1.02 (6.25) USD
Transport, food and accommodation (in outpatients’ services)	79.90 (247.03) USD	68.85 (94.25) USD	58.82 (57.10) USD	74.30 (199.42) USD
**Total direct non-healthcare costs**	**79.90 (247.03) USD**	**72.78 (96.82) USD**	**58.82 (57.10) USD**	**75.32 (199.71) USD**
Private health insurance reimbursement	3.94 (25.11) USD	35.71 (188.98) USD	5.71 (21.38) USD	12.41 (98.20) USD
**Total cost from a patient perspective (direct healthcare + direct non-healthcare–reimbursement)**	**799.96 (1929.33) USD**	**973.13 (1677.84) USD**	**284.11 (394.48) USD**	**777.99 (1741.45) USD**

### Cost estimation from a societal perspective

The mean cost per patient from a societal perspective was estimated at 23,803.21 USD (SD: 13,331.83) per patient. Direct healthcare costs reached 21,832.74 USD (SD: 13,217.98) per patient, with DMT being the main component of the total cost [97.3% (21,247.32 USD/patient)]. The direct non-healthcare costs represented 0.3% of the total direct cost (75.32 USD/patient). The indirect costs amounted to 8.0% of the total cost per patient and were estimated at 1,895.15 USD (SD: 2,479.59) per patient (Table 3).

**Table 3:** Cost estimation from the perspective of society according to disability level.

	EDSS 0–3.5(n = 66)	EDSS 3.5–6 (n = 28)	EDSS ≥6.5 (n = 14)	TOTAL (n = 108)
	Mean (SD)	Mean (SD)	Mean (SD)	Mean (SD)
***Direct healthcare costs***				
Consultations, examinations and treatments (in hospital)	0.00 (0.00) USD	25.18 (92.75) USD	0.00 (0.00) USD	6.52 (47.89) USD
Consultations, examinations and treatments (in outpatients’ services)	337.26 (1,119.33) USD	555.07 (1,377.01) USD	91.07 (191.92) USD	361.81 (1,224.18) USD
Additional examinations and tests	181.95 (543.38) USD	129.46 (359.63) USD	51.36 (123.34) USD	151.42 (464.61) USD
Drug treatment of flare-ups	27.89 (98.01) USD	54.94 (150.58) USD	4.23 (8.41) USD	31.83 (108.69) USD
Immunomodulatory drug treatment	22,635.19 (13,113.11) USD	21,387.86 (12,560.59) USD	14,423.43 (11,894.09) USD	21,247.32 (12,990.09) USD
Symptomatic drug treatment	38.88 (165.79) USD	29.93 (54.89) USD	17.80 (38.94) USD	33.83 (133.03) USD
**Total direct healthcare costs**	**23,221.17 (13,429.79) USD**	**22,182.44 (12,605.68) USD**	**14,587.89 (11,816.78) USD**	**21,832.74 (13,217.98) USD**
***Direct non-healthcare costs***				
Transport, food and accommodation (in hospital)	0.00 (0.00) USD	3.93 (11.96) USD	0.00 (0.00) USD	1.02 (6.25) USD
Transport, food and accommodation (in outpatients’ services)	79.90 (247.03) USD	68.85 (94.25) USD	58.82 (57.10) USD	74.30 (199.42) USD
**Total direct non-healthcare costs**	**79.90 (247.03) USD**	**72.78 (96.82) USD**	**58.82 (57.10) USD**	**75.32 (199.71) USD**
**Total direct costs**	**23,301.07 (13,379.68) USD**	**22,255.22 (12,658.54) USD**	**14,646.71 (11,822.96) USD**	**21,908.06 (13,201.98) USD**
***Indirect costs***				
Losses in the patient’s productivity	248.94 (1,785.72) USD	38.21 (125.02) USD	0.00 (0.00) USD	162.04 (1,397.56) USD
Losses in the productivity of family members	40.00 (307.95) USD	28.57 (108.38) USD	0.00 (0.00) USD	31.85 (246.47) USD
Losses due to long-term sick leave and early retirement	1,086.39 (1,935.21) USD	1,920.59 (2,258.40) USD	4,161.27 (1,197.70) USD	1,701.26 (2,184.92) USD
**Total indirect costs**	**1,375.33 (2,527.90) USD**	**1,987.37 (2,226.89) USD**	**4,161.27 (1,197.70) USD**	**1,895.15 (2,479.59) USD**
**Total cost from the perspective of society (direct healthcare +direct non-healthcare + indirect)**	**24,676.40 (13,725.49) USD **	**24,242.59 (12,873.83) USD **	**18,807.98 (12,066.20) USD **	**23,803.21 (13,331.83) USD **

Differences in costs were detected according to disability level. Cost was thus higher for patients with mild disability (24,676.40 USD /patient), followed by patients with moderate disability (24,242.59 USD/patient) and, lastly, patients with severe disability (18,807.98 USD/patient) (Table 3). The losses in productivity were lower for the latter since they were not patients in a situation of active employment.

## Discussion

COI studies are a very useful tool for identifying, quantifying and assessing all the economic resources involved in decision-making regarding the health-disease-care process [40]. An awareness of the size of the resources assigned for a disease, as well as the resources lost through premature morbi-mortality, is essential for the design and implementation of health sector healthcare policies.

This work is the first COI study of MS undertaken in Panama. It uses a bottom-up method and prevalence approach, both of which are the most frequently used methodologies in studies on the costs of MS [41].

Study results show that, although the prevalence of MS is low, the disease imposes a significant economic burden both for patients and for society, which reaches 777.99 USD/patient and 23,803.21 USD/patient, respectively. The cost of drugs associated with DMT is one of the main components of total cost per patient in all phases of the disease. The components of the total cost vary according to the disease stage. Thus, in patients with mild disability (EDSS 0–3.5) main component of total cost is related to immunomodulatory drug treatment, while direct cost associated to hospitalization or relapses treatment, and indirect cost are lower since flare-ups are less frequent and work productivity is less compromised. Total cost associated to patients with moderate disability (EDSS 3.5–6) is the highest, mainly due to pharmacological cost (immunomodulatory drug treatment, drugs treatment for flare-ups and symptomatic drug treatment) and direct cost related to health care resources use (in hospital and in outpatients’ services). Moreover, indirect cost associated to these patients is also high. Finally, since most patients in more advanced stages do not receive DMT drugs but, rather, drugs with a lower associated cost, the cost of drugs at this stage is lower. In patients with severe disability, the loss in work productivity is considerable. These patients were the population group with a greater associated indirect cost.

The results obtained in this study are similar to those of studies performed in Latin America and in Europe, with a few differences [7,19,41,42]. Thus, the results obtained in a cross-sectional study performed in 16 European countries (Austria, Belgium, Czech Republic, Denmark, France, Germany, Hungary, Italy, Netherlands, Poland, Russia, Spain, Sweden, Switzerland and the United Kingdom) also showed that MS costs were related to disease severity (EDSS score) and were dominated by production losses, non-healthcare cost and DMTs [19]. The results in the 16 countries depended very much on the composition of the sample in terms of age, disease severity and employment status. While total costs per patient were similar across countries for participants with mild MS (EDSS <3), as MS progressed and became more severe, disparities between countries appeared.

In our study, patients with the highest associated cost are those with mild and moderate disability, while patients with severe disability generate a lower cost. It is therefore important to bear in mind that in severely disabled patients DMT are less frequent, which reduces their associated cost by approximately 20%. This may reflect differences in healthcare organisations, medical traditions, ease of access and availability of given services, since in Panama, community assistance such as nurse visits, home help, neuro-rehabilitation services, social and welfare institutions for chronic disease are scarce. It is also important to consider that these data should be analysed with caution, as patients with severe disability represent a small number of the total sample (n = 14) and the results may not be representative of this subgroup of patients.

In addition to data on costs, this study provides very useful information on the HRQoL of MS patients in Panama. HRQoL assessment shows a clear deterioration in the HRQoL of these patients due to the disease. The pain/discomfort, mobility and anxiety/depression dimensions are those most affected, while the self-care dimension is less affected by MS. The results of our study show that severely disabled patients have a HRQoL score of 25 fewer points (VAS 0–100 scale) than patients with mild disability, which represents significant deterioration in HRQoL. The mobility and usual activities dimensions of HRQoL are those most affected by the disability’s progression, while the anxiety/depression dimension is affected in all stages of the disease. These results are consistent with previous studies [7,28,43], suggesting that therapies that can slow down MS progression will also help to reduce the deterioration in HRQoL that accompanies this disease.

Since, Panama national health system covers MS related costs, this study provides relevant information related to the impact of MS disability progression in both cost and patients’ HRQoL, useful for the public authorities to substantiate that the considerable investment in DMTs represents an efficient use of public funds. Since both, direct and indirect cost associated to patients with moderate disability (EDSS 3.5–6) are high, developing new health policies in the region in order to improve MS diagnostic and patients access to MS treatment may contribute to reduce total cost associated to MS promoting the early diagnosis and early access to treatment.

The study has certain limitations, many of which are inherent in COI studies [41]. The main limitation is the availability of data with which to estimate costs in the most severe cases. The inclusion of costs no available in this group of patients may increase the total cost per patient.

## Conclusion

The results of this study provide up-to-date data on the cost and on the HRQoL of MS patients in Panama. The cost per MS patient is high, both from societal and patient perspectives. Main component of the cost of patients with mild disability is associated with the cost of immunomodulatory therapy. Patients with moderate disability accomplish the highest cost mainly due to a high pharmaceutical cost, cost related to use of healthcare resources and indirect cost. Finally, while indirect cost is higher in patients with severe disability, direct cost is lower. In addition to the high economic impact, MS has a negative influence on patient HRQoL, which increases as the disease progresses.

Further research is required to explore the precise economic burden of severely-disabled patients and in the most advanced stages of the disease.
